# Heterogeneous relationships of squamous and basal cell carcinomas of the skin with smoking: the UK Million Women Study and meta-analysis of prospective studies

**DOI:** 10.1038/s41416-018-0105-y

**Published:** 2018-06-14

**Authors:** Kirstin Pirie, Valerie Beral, Alicia K. Heath, Jane Green, Gillian K. Reeves, Richard Peto, Penelope McBride, Catherine M. Olsen, Adèle C. Green

**Affiliations:** 1 Cancer Epidemiology Unit, Nuffield Department of Population Health, University of Oxford, Oxford, UK; 2 Population Health Research Unit, Nuffield Department of Population Health, University of Oxford, Oxford, UK; 3 Population Health Department, QIMR Berghofer Medical Research Institute, Brisbane, QLD Australia; 4 School of Public Health, University of Queensland, Brisbane, QLD Australia; 5 Cancer Research UK Manchester Institute and Institute of Inflammation and Repair, University of Manchester, Manchester, UK

**Keywords:** Basal cell carcinoma, Squamous cell carcinoma, Risk factors, Cancer epidemiology

## Abstract

**Introduction:**

Published findings on the associations between smoking and the incidence of cutaneous squamous cell carcinoma (SCC) and basal cell carcinoma (BCC) are inconsistent. We aimed to generate prospective evidence on these relationships overall and by anatomical site.

**Methods:**

We followed 1,223,626 women without prior cancer by electronic linkage to national cancer registration data. Questionnaire information about smoking and other factors was recorded at recruitment (1996–2001) and every 3–5 years subsequently. Cox regression yielded adjusted relative risks (RRs) comparing smokers versus never-smokers.

**Results:**

After 14 (SD4) years follow-up per woman, 6699 had a first registered cutaneous SCC and 48,666 a first BCC. In current versus never-smokers, SCC incidence was increased (RR = 1.22, 95% CI 1.15–1.31) but BCC incidence was decreased (RR = 0.80, 0.78–0.82). RRs varied substantially by anatomical site; for the limbs, current smoking was associated with an increased incidence of SCC (1.55, 1.41–1.71) and a decreased incidence of BCC (0.72, 0.66–0.79), but for facial lesions there was little association of current smoking with either SCC (0.93, 0.82–1.06) or BCC (0.92, 0.88–0.96). Findings in meta-analyses of results from this and seven other prospective studies were largely dominated by the findings in this study.

**Conclusions:**

Smoking-associated risks for cutaneous SCC and BCC are in the opposite direction to each other and appear to vary by anatomical site.

## Introduction

Although rarely fatal, cutaneous squamous cell and basal cell carcinomas (SCC and BCC, respectively) are among the most common types of cancer in sun-exposed white populations.^1,2^ Excessive sun exposure is known to be an important cause of both,^3^ but evidence about the associations with smoking is inconsistent and smoking-related risks may vary by tumour histology and anatomical site.^4–6^ Two meta-analyses published in 2012 concluded that smokers had a slightly increased risk of SCC but not of BCC.^5,7^ Since then, however, three reports suggested a decreased risk of BCC in current smokers.^6,8,9^

To provide reliable epidemiological evidence about the associations between smoking and the incidence of cutaneous SCC and BCC, overall and by anatomical site, we present new data from a large prospective study of UK women, and we update previous meta-analyses.

## Materials and methods

In 1996–2001, participants were recruited into the Million Women Study through the National Health Service (NHS) Breast Screening Programme and gave signed consent for follow-up when completing a questionnaire about lifestyle, medical and socio-demographic factors. Ethical approval was from Oxford and Anglia MREC. Further details, including study questionnaires and data access policy, can be found on the study website (http://www.millionwomenstudy.org).^10^

People in the UK have unique NHS numbers that enable electronic linkage to the NHS Central Registers, through which researchers can be routinely notified of cancer registrations and deaths. SCCs and BCCs can occur more than once in the same person, and in England the recommendation is to register the first SCC and the first BCC in an individual, whereas in Scotland the recommendation is to register all SCCs but only the first BCC.^11^ Cancer site and morphology are coded to the International Classification of Diseases ICD-10 and ICD-O-3.^12,13^ The main outcomes are SCC (ICD-10 C44, ICD-O-3 8070/3-8076/3) and BCC of the skin (ICD-10 C44, ICD-O-3 8090/3-8097/3, including nodular [8097/3], superficial [8092/3] and infiltrating [8091/3] subtypes). These are further classified by anatomical site, determined by the fourth character of the ICD-10 code: lip (C44.0), face including eyelid (C44.1, C44.3), ear, scalp and neck (C44.2, C44.4), trunk (C44.5), upper limb (C44.6), lower limb (C44.7), overlapping lesion or unspecified site (C44.8 or C44.9). The small number of women (*n* = 120) who had a first SCC and a first BCC registered on the same date are included as a case for each outcome; for analyses by anatomical site, women with overlapping lesions or cancers of unspecified site were excluded, as were the few women who had more than one site specified on the same date (*n* = 3 for SCC and *n* = 2 for BCC). As ICD-10 code C00 is for malignant neoplasm of the lip (excluding the skin of lip), results are also presented for cancers of the lip based on either ICD-10 code (C00 or C44.0).

At recruitment, which is the baseline for the main analyses, women were asked if they were a current smoker or an ex-smoker and how many cigarettes they now smoked (in categories: none, <5, 5–9, 10–14, 15–19, 20–24, 25+ cigarettes/day). Study participants were resurveyed by post about every 3–5 years after recruitment, each time asking about current and past smoking. In addition, women were asked at the resurvey 8 (SD 3) years after recruitment their hair colour when aged 10 years, and at the resurvey 12 (SD 2) years after recruitment they were asked about their eye colour, number of freckles and moles, sunbed use, holidays in sunny places and tendency to tan or burn.

### Statistical analysis

The effects of smoking were assessed for each anatomic site, and for any site, for the first incident SCC and first incident BCC. Because smoking and other behaviours might change after cancer diagnosis, women were excluded if, at recruitment, they had a prior registration of any invasive cancer, including cutaneous SCC or BCC (*n* = 54,663). Women with unknown smoking status (*n* = 10,533) and non-smokers whose incomplete replies did not allow us to distinguish reliably between being never-smokers and ex-smokers (*n* = 65,730) were also excluded. The remaining 1,223,626 women contributed person-years until 1.1.2015 or until their first registration of SCC, BCC or any other invasive cancer, death or emigration, irrespective of whether they responded to subsequent resurveys. The few women (1.5%; 18,226/1,223,626) who left the UK permanently or ceased to be registered with a general practitioner were lost to follow-up on the date they did so.

For the main analyses, Cox proportional hazards models (with time in the study as the underlying time variable) yielded adjusted incidence rate ratios (RRs, also referred to as relative risks) that compare various categories of smokers or ex-smokers to never-smokers, using smoking status reported at recruitment. Categories of amount smoked were defined using information reported at recruitment. Results were stratified by year of birth, year of recruitment and geographical region (Scotland, and nine cancer registry regions in England) and adjusted for socio-economic status (quintiles of 1991 Townsend deprivation index for area of residence at recruitment),^14^ current alcohol intake (none, ≤7, >7 drinks/week), strenuous physical activity (rarely/never, at least once a week), height (<160, 160–164.9, ≥165 cm) and body mass index (<20, 20–24.9, 25–29.9, ≥30 kg/m^2^). For each variable, missing values formed a separate category.

To assess the effect of potential confounding by changes in smoking and by hair colour, eye colour, freckles, moles, tendency to tan or burn, regular sunbed use and recent sun exposure, analyses were repeated among the 486,493 study participants who responded to the 12-year resurvey and had known smoking status and no prior cancer (including SCC or BCC). Cox regression yielded RRs of SCC and BCC associated with smoking as reported at the 12-year survey, adjusting for hair colour at age 10 years (blonde, red, brown, black, other), eye colour (blue, grey, brown, green, hazel, other), number of moles (about average, more, fewer) and freckles (about average, more, fewer), regular sunbed use (no, <5, ≥5 years of use), number of holidays in sunny places in the last 5 years (none, ≤5, >5) and propensity to burn (burn easily, tan easily, rarely tan) as well as for all factors described in the paragraph above. All adjustment variables were based on responses to the 12-year survey, apart from hair colour which used information from the 8-year survey. Tests for interaction between smoking and each variable listed above were done using likelihood ratio tests, comparing models with and without an interaction term.

Where RRs are plotted as squares, the area of each square is inversely proportional to the variance of the log RR, and corresponding 95% confidence intervals (CIs) are plotted as lines. All calculations used Stata version 14.1.

### Meta-analysis of prospective studies

Relevant publications from prospective studies were identified by reviewing articles and by a literature search using PubMed, up to June 30, 2017 (The systematic search strategy and findings from it are given in Supplementary Information). RRs and 95% CIs comparing current smokers or ex-smokers versus never-smokers were extracted from prospective studies (i.e. studies where information on smoking was recorded prior to cancer diagnosis) for incident SCC and for incident BCC separately. Summary RRs, combining study-specific results, were calculated as weighted averages, with each weight proportional to the inverse of the variance of the study-specific log RR. Chi-squared tests assess heterogeneity across studies.

### Role of funding sources

Sponsors had no role in study design, data collection, data analysis, data interpretation or report writing.

## Results

After exclusion of women with previous cancer and unknown smoking status 1,223,626 remained, on average born in 1943 (interquartile range 1938–1946) and recruited in 1998 (range 1996–2001) at age 56 (SD 5) years. Women were followed for an average of 14.4 (SD 4) years, during which 0.5% (6699) had a first registration of cutaneous SCC, at mean age 69 (SD 6) years, and 4.0% (48,666) had a first registration of cutaneous BCC, at mean age 67 (SD 6) years. The mean time from recruitment to diagnosis was 10 (SD 5) years.

At recruitment, 21% (252,547), 28% (346,657) and 51% (624,422), respectively, reported that they were current smokers, ex-smokers and never-smokers. Their characteristics are compared in Table 1. Current smokers were more likely than never-smokers to live in deprived areas, drink >7 units of alcohol per week and do no strenuous exercise. Among women who completed the resurvey questionnaires asking about sun exposure and sensitivity to the sun, current smokers were more likely than never-smokers to report that they tanned easily and to have ever regularly used a sunbed, whereas there was little difference by eye or hair colour. Additional adjustments for these factors were done in sensitivity analyses among women who completed the 12-year resurvey (see below).

**Table 1:** Characteristics of study participants by smoking status reported at recruitment

	Never-smoker	Ex-smoker	Current smoker
	*N* = 624,422	*N* = 346,657	*N* = 252,547
Characteristics reported at recruitment			
Age, years	56.3 (4.7)	56.1 (4.6)	55.3 (4.3)
Socio-economic status, highest third	38.0 (235,471)	33.3 (114,536)	22.6 (56,592)
Height, cm	162.1 (6.6)	162.4 (6.6)	161.6 (6.7)
Body mass index, kg/m^2^	26.2 (4.6)	26.7 (4.8)	25.6 (4.5)
Any strenuous exercise	55.0 (333,709)	53.1 (178,715)	40.2 (95,943)
More than 7 units of alcohol per week	14.4 (89,725)	25.9 (89,340)	23.7 (59,010)
Characteristics reported at resurveys			
Red hair when aged 10 years^a^	4.7 (16,142)	4.9 (8893)	5.1 (4773)
Blue eyes^b^	39.0 (113,585)	38.6 (58,620)	39.1 (29,072)
More freckles than average^b^	12.7 (33,944)	12.7 (17,716)	11.0 (7,252)
More moles than average^b^	13.4 (32,961)	13.1 (16,716)	12.6 (7,511)
Burn easily^b^	42.3 (122,417)	41.9 (63,344)	36.5 (26,954)
Tan easily^b^	41.5 (120,212)	44.4 (67,229)	48.6 (35,913)
>5 holidays in sunny places in the past 5 years^b^	24.5 (68,411)	27.4 (39,934)	24.2 (17,108)
Ever regularly used a sunbed^b^	6.7 (17,676)	10.8 (14,734)	10.5 (6899)
Follow-up from recruitment			
Average years of follow-up per woman	14.6 (3.7)	14.4 (3.9)	13.9 (4.2)
Number developing an incident BCC	26,996	14,312	7358
Number developing an incident SCC	3509	1857	1333

Data are percentage of women (*n*) or mean (SD) unless otherwise specified.

*BCC* basal cell carcinoma, *SCC* squamous cell carcinoma.

^a^Reported at mean 7.8 years after recruitment by 619,521 women.

^b^Reported at mean 12.5 years after recruitment by 531,066 women

Table 2 shows RRs for first incident SCC and for first incident BCC by smoking category at recruitment. Current smokers had about a 20% higher incidence of cutaneous SCC than never-smokers (adjusted RR = 1.22, 95% CI 1.15–1.31) and a 20% lower incidence of BCC than never-smokers (adjusted RR = 0.80, 0.78–0.82). Among current smokers, the incidence of SCC was greater in those who smoked >15 cigarettes per day than those who smoked fewer (test of dose dependence among current smokers: *p* = 0.003). For BCC, there was no significant relationship with the amount smoked (*p* = 0.9). There was little or no association of skin cancer incidence with past smoking, either for SCC (RR = 1.00, 0.95–1.06) or for BCC (RR = 0.99, 0.97–1.01).

**Table 2:** Relative risk (RR) of squamous cell and basal cell carcinoma of the skin by smoking status reported at recruitment

	Squamous cell carcinoma		Basal cell carcinoma	
	*N*	RR (95% CI)	*N*	RR (95% CI)
All cutaneous lesions				
Never-smoker	3509	1.00	26,996	1.00
Ex-smoker	1857	1.00 (0.95–1.06)	14,312	0.99 (0.97–1.01)
Current smoker	1333	1.22 (1.15–1.31)	7358	0.80 (0.78–0.82)
<15 cigarettes/day	682	1.14 (1.05–1.24)	3976	0.80 (0.77–0.83)
≥15 cigarettes/day	651	1.34 (1.23–1.46)	3382	0.80 (0.77–0.83)
Lesions on the face				
Never-smoker	1097	1.00	9525	1.00
Ex-smoker	534	0.93 (0.84–1.04)	5136	0.99 (0.96–1.03)
Current smoker	317	0.93 (0.82–1.06)	3193	0.92 (0.88–0.96)
<15 cigarettes/day	151	0.80 (0.68–0.95)	1662	0.90 (0.85–0.95)
≥15 cigarettes/day	166	1.09 (0.92–1.29)	1531	0.94 (0.89–0.99)
Lesions on the limbs				
Never-smoker	1437	1.00	2657	1.00
Ex-smoker	798	1.05 (0.96–1.15)	1409	0.98 (0.92–1.05)
Current smoker	663	1.55 (1.41–1.71)	652	0.72 (0.66–0.79)
<15 cigarettes/day	358	1.50 (1.34–1.69)	362	0.74 (0.66–0.83)
≥15 cigarettes/day	305	1.61 (1.42–1.83)	290	0.70 (0.62–0.79)

For women with a first registered SCC, a single specific anatomical site was recorded for 91% (6078/6699). Of these lesions, 32% (1947/6078) were on the face and 48% (2895/6078) on the limbs. For women with a first registered BCC, a single specific anatomical site was recorded for 61% (29,594/48,666), and of these lesions 60% (17,852/29,594) were on the face and 16% (4716/29,594) on the limbs. Tumour distributions by anatomical site are similar to those for women aged 45–74 years in England in 2008–2010 (Supplementary Table 1).

**Fig. 1:**
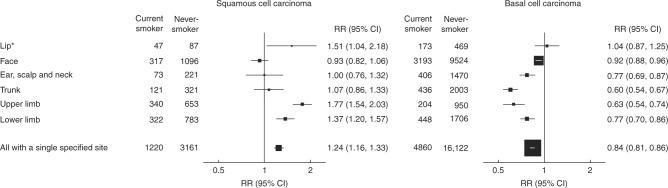
Relative risk of squamous cell and basal cell carcinoma of the skin for the current smokers versus never-smokers by anatomical site. *ICD-10 C44.0 only: 19 current smokers and 33 never-smokers had a squamous cell carcinoma (scc) coded to ICD-10 C00 (malignant neoplasm of lip, excluding skin of lip): combined RR for SCC of the lip (ICD-10 C00 or C44.0) =1.55 (95% CI 1.13–2.12). Only 1 current smoker and 1 never-smoker had a basal cell carcinoma coded to ICD-10 C00

The overall RRs for current versus never-smokers for tumours with a single specified site recorded were similar to those for all SCCs and BCCs (Fig. 1 and Table 2). There was, however, considerable variation in the smoking-related risks by anatomical site, both for SCC and BCC (test for heterogeneity by site *p* < 0.001 for each type, Fig. 1). For limb lesions, the RRs for current compared to never-smokers were substantially different for SCCs and BCCs (1.55, 1.41–1.71 for limb SCCs and 0.72, 0.66–0.79 for limb BCCs). For trunk lesions, the RRs were again different for SCCs and for BCCs (1.07, 0.86–1.33 for trunk SCCs and 0.60, 0.54–0.67 for trunk BCCs). By contrast, for facial lesions the RRs comparing current versus never-smokers appeared to be similar for SCCs and for BCCs (0.93, 0.82–1.06 for facial SCCs and 0.92, 0.88–0.96 for facial BCCs).

Histology was non-specific for 99% (6668) of SCCs and 93% (45,468) of BCCs. Of the BCCs with a specified subtype, 49% (1577) were nodular, 23% (735) were superficial and 21% (664) were infiltrating tumours; where anatomical site was also specified, most nodular and infiltrating BCCs occurred on the head and neck (80% [1254/1569] and 83% [511/614], respectively) compared to just 34% of superficial BCCs (232/688). The RR for current versus never-smokers was more marked for superficial BCCs (0.54, 0.43–0.69) than for nodular and infiltrating subtypes (0.79, 0.70–0.91 and 0.83, 0.67–1.04, respectively; Supplementary Figure 1).

The current smoker versus never-smoker RRs for cutaneous SCC and for BCC of the lip are plotted in Fig. 1 for cancers registered as ICD-10 C44.0 (other malignant neoplasm of skin of lip) and do not include cancers registered as ICD-10 C00 (malignant neoplasm of lip, excluding skin). Smoking is associated with an increased risk of SCC registered as C44.0 (1.51, 1.04–2.18) and also with an increased risk of SCC registered as either C44.0 or C00 (1.55, 1.13–2.12). As there were relatively few cases of lip cancer, the RRs for all SCCs and for all BCCs remain essentially unchanged if cancers of the lip are excluded (1.22, 1.14–1.30 for all SCCs and 0.80, 0.78–0.82 for all BCCs).

All analyses were routinely adjusted for the ten UK cancer registration regions that were in place when the women were recruited: one covering Scotland and 9 in England (although since then two English registries have merged).^15^ Registration rates for BCC have been reported to be low in the Thames region,^11^ perhaps because of under-registration, and consistent with the national statistics, BCC registration rates in this cohort were lowest in the Thames region but did not vary substantially across the other regions (Supplementary Table 2). Nevertheless, the RRs comparing current versus never-smokers did not vary significantly by region for SCC or for BCC and were therefore unchanged by excluding the Thames region (Supplementary Figure 2).

**Fig. 2:**
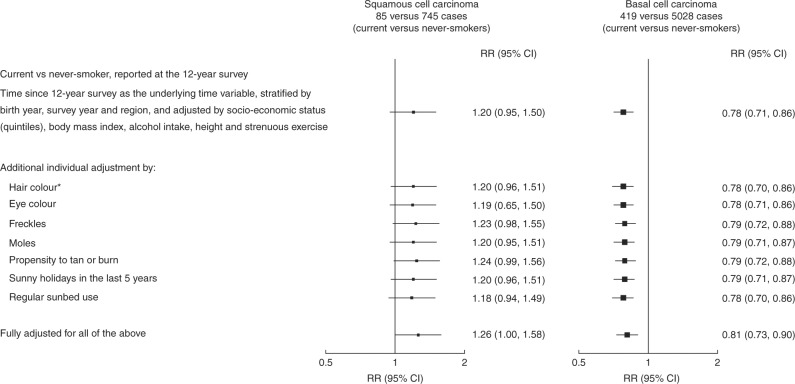
Relative risk of squamous cell carcinoma and basal cell carcinoma of the skin for current smokers versus never-smokers as reported at the 12-year survey, with various adjustments. *Hair colour reported at the 8-year survey only; 23 % (113,337/486,493) had no available information on hair colour

To assess the role of confounding by hair and eye colour and by various measures of sun exposure and sensitivity to the sun, the main analyses were repeated among 486,493 women who provided information on these factors at the 12-year resurvey in 2009–2012 (and had no prior cancer at this time; Fig. 2). Women who completed this resurvey were aged 68 (SD 5) years on average, and during 3.5 (SD 1) years of follow-up, 1343 had a first registration of cutaneous SCC, at a mean age 72 (SD 5) years, and 8612 had a first registration of cutaneous BCC, at a mean age 71 (SD 5) years. At the 12-year survey, 7% (32,909), 38% (184,938) and 55% (268,646), respectively, reported that they were current smokers, ex-smokers and never-smokers. The RRs for incident SCC and BCC in current compared to never-smokers were similar to those in the entire cohort (1.20 versus 1.22 for SCC and 0.78 versus 0.80 for BCC), although with the smaller numbers the excess of SCC was not significant. After additional adjustment for hair colour, eye colour, moles, freckles, tendency to burn or tan, regular sunbed use and number of recent holidays in sunny places, the excess of SCC and significant deficit of BCC both remained, but the non-significant RR for SCC was slightly increased (from 1.20 to 1.26) and the significant RR for BCC was slightly attenuated (from 0.78 to 0.81). There were no significant interactions between smoking and any of the markers of sun exposure or sensitivity to the sun with BCC or SCC risk (*p* > 0.1 for each).

**Fig. 3:**
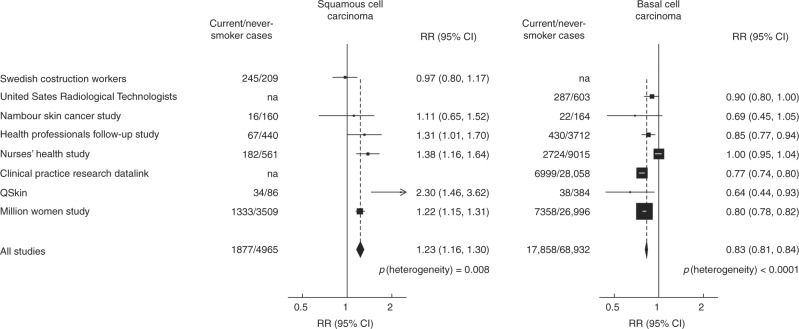
Meta-analysis of prospective studies for current smokers versus never-smokers. *na* not available

In our systematic review, we identified seven other prospective studies^4–6,8,9,16,17^ that had reported on the risk of incident cutaneous SCC or BCC separately in current smokers and in ex-smokers versus never-smokers (Supplementary Figure 3). Taking all studies together, including the present study, the combined RRs for SCCs and BCCs comparing current smokers versus never-smokers were similar to those in the current study (1.23, 1.16–1.30 for SCC and 0.83, 0.81–0.84 for BCC), but there was significant heterogeneity across studies (*p* = 0.008 for SCC and *p* < 0.001 for BCC; Fig. 3). For ex-smokers versus never-smokers, there were no significant associations either for SCC or for BCC (Supplementary Figure 4).

## Discussion

We found that current smokers were at slightly increased overall risk of cutaneous SCC but at slightly reduced overall risk of cutaneous BCC. There was also substantial heterogeneity between the RRs for SCCs on different parts of the body and between the RRs for BCCs on different parts of the body. About a third of the cutaneous SCCs and two thirds of the cutaneous BCCs with a specified site were on the face, and smoking appeared to have little association with either (RRs 0.93 for facial SCC and 0.92 for facial BCC). By contrast, for lesions on the limbs current smoking was associated with a significantly increased risk for SCC and a significantly decreased risk for BCC (RRs 1.55 and 0.72, respectively; *p* for heterogeneity <0.001). The distributions of SCCs and of BCCs on different parts of the body in women in this study are similar to those reported in national cancer registry statistics for all women aged 45–74 years in England (Supplementary Table 2). As this study includes women only, results may not be generalisable to men.

Meta-analysis of the results from this and seven other prospective studies also showed opposite associations of smoking with SCCs and with BCCs but were largely dominated by the results of this study. Only two other reports gave results for BCC by anatomical site and their findings are consistent with those reported here.^6,8^

Most UK cancer registries record just the first SCC and just the first BCC for any individual, and our analyses therefore used first registered SCC and first registered BCC as the main outcomes. Registration of BCC and SCC is estimated to be 10–30% incomplete in England, with under-registration of BCC a particular problem in the Thames region.^11,18^ This was confirmed in the Million Women Study, where BCC rates in the Thames region were lower than in other cancer registry regions. Nevertheless, we took account of different cancer registration rates in all analyses by routinely stratifying by the 10 cancer registries in England and Scotland that existed at time of recruitment, and the smoking-associated RRs did not vary significantly by region or on excluding the Thames region (Supplementary Figure 2).

As well as stratifying by cancer registry region, all results were adjusted for the information available to us on eight other potential confounders: year of birth, deprivation index, height, body mass index, alcohol consumption, strenuous exercise, year of recruitment, and time since recruitment. In the subset who answered questions after recruitment about hair colour, eye colour, moles, freckles, regular sunbed use, recent holidays in sunny places and tendency to burn or tan, the associations of smoking with SCCs and with BCCs were not materially altered after additional adjustment by each factor separately and for all factors simultaneously.

The most striking findings of this study are the opposite smoking-associated RRs by tumour type, with SCCs positively associated and BCCs negatively associated with smoking, and the substantial variation between anatomical sites in the magnitudes of these two associations. Residual confounding would generally be expected to bias smoking-related risks in a similar way for both SCC and BCC but not lead to substantial heterogeneity. Residual confounding thus seems unlikely to account for these strongly divergent smoking-associated risks, particularly for lesions of the limbs, where there are substantial excesses for SCC and substantial deficits for BCC (RRs of 1.6 versus 0.7). Differential cancer registration again seems unlikely to explain these differences, as cancer registries would have had to have differentially recorded site-specific cutaneous cancers in current smokers.

It is possible, however, that health-seeking never-smokers may be more likely than current smokers to have their skin checked, which would result in lower incidence of BCC in smokers, particularly for less visible anatomical sites where the tumour may have otherwise have gone unnoticed,^19^ but this would not explain the increased incidence for SCC of the limbs in smokers compared to never-smokers. Limbs are often covered by clothing and are less regularly exposed to ultraviolet (UV) radiation than sun-exposed parts of the body like the face, and so it may be that smoking is associated with SCC only at lower levels of UV exposure. However, there were no significant interactions between smoking and any of the markers of sun exposure, sunbed use or sensitivity to the sun in this study (*p* > 0.1 for each).

It is well established that smoking-related risks for cancers of other organs can vary by tumour type. Furthermore, smoking has generally been found to cause greater increases in the incidence of SCC than of other types of carcinoma, similar to the findings here for skin cancer. For example, smoking has a significantly greater effect for SCCs than adenocarcinomas of the lung,^20^ of the cervix^21^ and of the anus.^22^ For BCC, a greater deficit in smoking-related risk was observed for superficial BCCs (RR 0.54, 0.43–0.69), which occur more commonly on the trunk and limbs, than for nodular BCCs (RR 0.79, 0.70–0.91), which occur more frequently on the head,^23^ but as histological subtype was not specified for 93% of BCCs these results should be interpreted with caution.

In conclusion, smoking-associated RRs for SCC and BCC of the skin are heterogeneous, with risks varying both by tumour type and by anatomical site. There appears to be little or no association of current smoking with the incidence of SCC and of BCC of the face. By contrast, current smokers have increased incidence of SCC of the limbs but reduced incidence of BCC of the limbs and trunk. While sun exposure is an important cause of both types of cutaneous cancer, current smoking is also associated with the risk of these cancers.

## Electronic supplementary material

Supplementary material

## Availability of data and materials

Information on data access for the Million Women Study is available at www.millionwomenstudy.org/data_access/.
