# Supplementary material for: Heterogeneous relationships of squamous and basal cell carcinomas of the skin with smoking: the UK Million Women Study and meta-analysis of prospective studies
Source: Br J Cancer. 2018 Jun 14;119(1):114–20. doi: 10.1038/s41416-018-0105-y (PMC6035218; doi:10.1038/s41416-018-0105-y)
Supplement: Supplementary file 1 — Supplementary material [file 41416_2018_105_MOESM1_ESM.docx]

**Heterogeneous relationships between smoking and squamous and**

**basal cell carcinomas of the skin: prospective study of UK women and meta-analysis**

Kirstin Pirie^1^ MSc, Prof Valerie Beral^1^ FRS, Alicia K Heath^1^ PhD, Prof Jane Green^1^ DPhil,

Prof Gillian K Reeves^1^ PhD, Prof Richard Peto^2^ FRS, Penelope McBride^3^ MBBS,

Catherine M Olsen^3,4^ PhD, Prof Adèle C Green^3,5^ MBBS

^1^Cancer Epidemiology Unit, Nuffield Department of Population Health, University of Oxford, Oxford, UK; ^2^Clinical Trial Service Unit and Epidemiological Studies Unit (CTSU), Nuffield Department of Population Health, University of Oxford, Oxford, UK; ^3^Population Health Department, QIMR Berghofer Medical Research Institute, Brisbane, Australia; ^4^School of Public Health, University of Queensland, Brisbane, Australia; ^5^Cancer Research UK Manchester Institute and Institute of Inflammation and Repair, University of Manchester, Manchester Academic Health Science Centre, Manchester, UK

**Short title:** Smoking and cutaneous BCC and SCC

**Abbreviations:** **SCC** Squamous cell carcinoma; **BCC** Basal cell carcinoma

**Supplementary information**

**Supplementary Table 1.** Distribution of squamous cell and basal cell carcinomas of the skin by anatomical site in the Million Women Study, 1996-2014, and in Cancer Registry data for England, 2008-2010* among those with a specific site recorded.

|  | **Face, ear, scalp and neck** | **Upper and lower limbs** | **Trunk** | **Total, N**  **(=100%)** |
| --- | --- | --- | --- | --- |
| **Squamous cell carcinoma** |  |  |  |  |
| Million Women Study | 42% (2536) | 48% (2895) | 11% (647) | 6078 |
| National registry data* | 40% (2416) | 46% (2762) | 13% (807) | 5985 |
|  |  |  |  |  |
| **Basal cell carcinoma** |  |  |  |  |
| Million Women Study | 72% (22,433) | 16% (4716) | 12% (3445) | 29,594 |
| National registry data* | 71% (20,533) | 15% (4366) | 14% (4017) | 28,916 |

*Cancer registration data for England, females aged 45-74 years, 2008-10.

**Supplementary Table 2.** Cancer registration rates for squamous cell and basal cell carcinomas of the skin per 100,000 women per year, by cancer registration region.

| **UK Cancer Registry Region** | **Squamous cell carcinoma,**  **rate per 100,000 (n)** | **Basal cell**  **carcinoma,**  **rate per 100,000 (n)** |
| --- | --- | --- |
| South West | 49 (1810) | 321 (11,945) |
| Thames | 37 (905) | 193 (4696) |
| Oxford | 37 (429) | 322 (3694) |
| Eastern | 34 (309) | 297 (2666) |
| West Midlands | 31 (483) | 270 (4232) |
| Trent | 38 (783) | 268 (5531) |
| North West (Mersey) | 44 (509) | 298 (3442) |
| North West (Manchester/Lancashire) | 34 (438) | 270 (3468) |
| Northern & Yorkshire | 29 (548) | 285 (5407) |
| Scotland | 34 (485) | 248 (3585) |

**
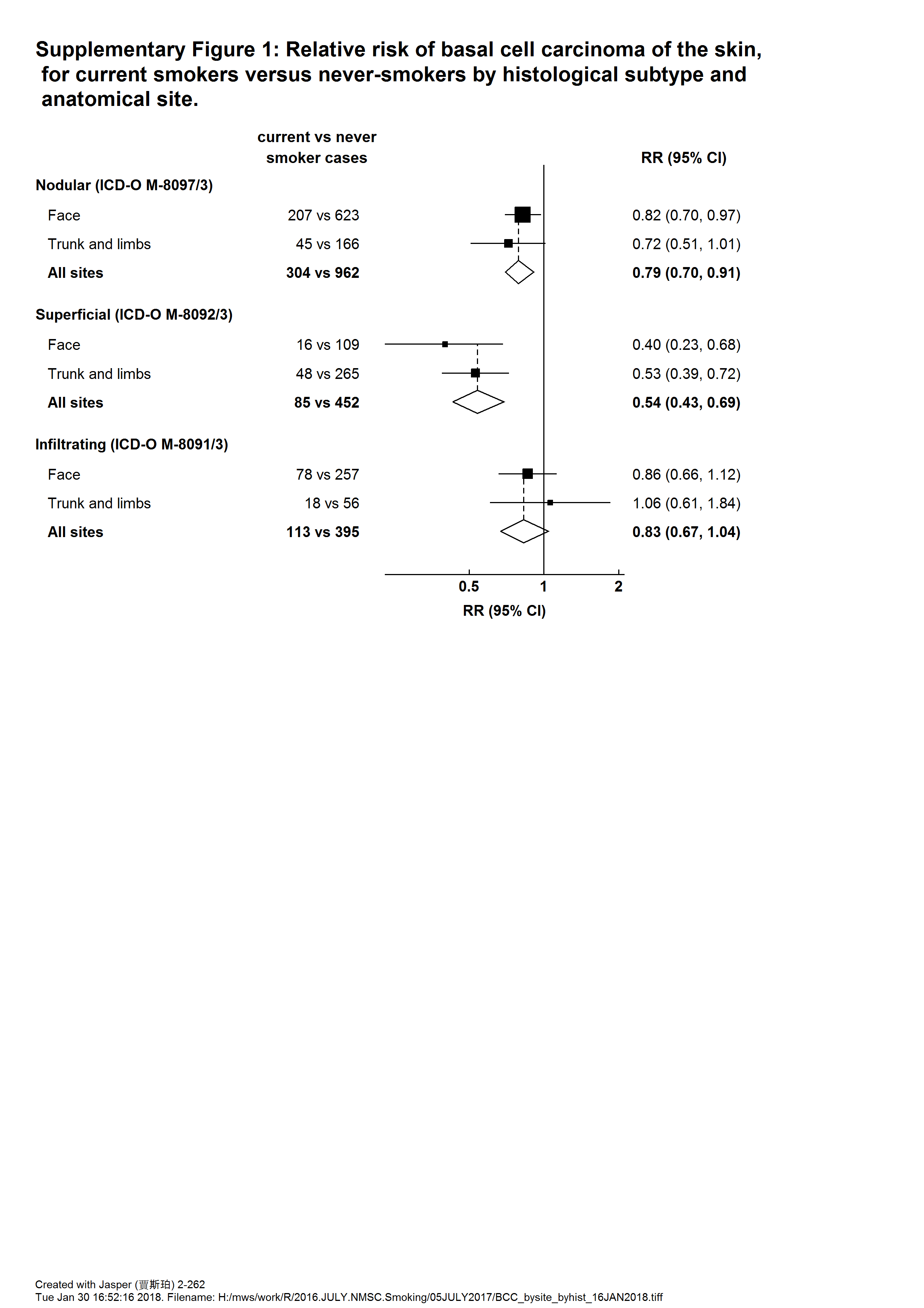
**

**
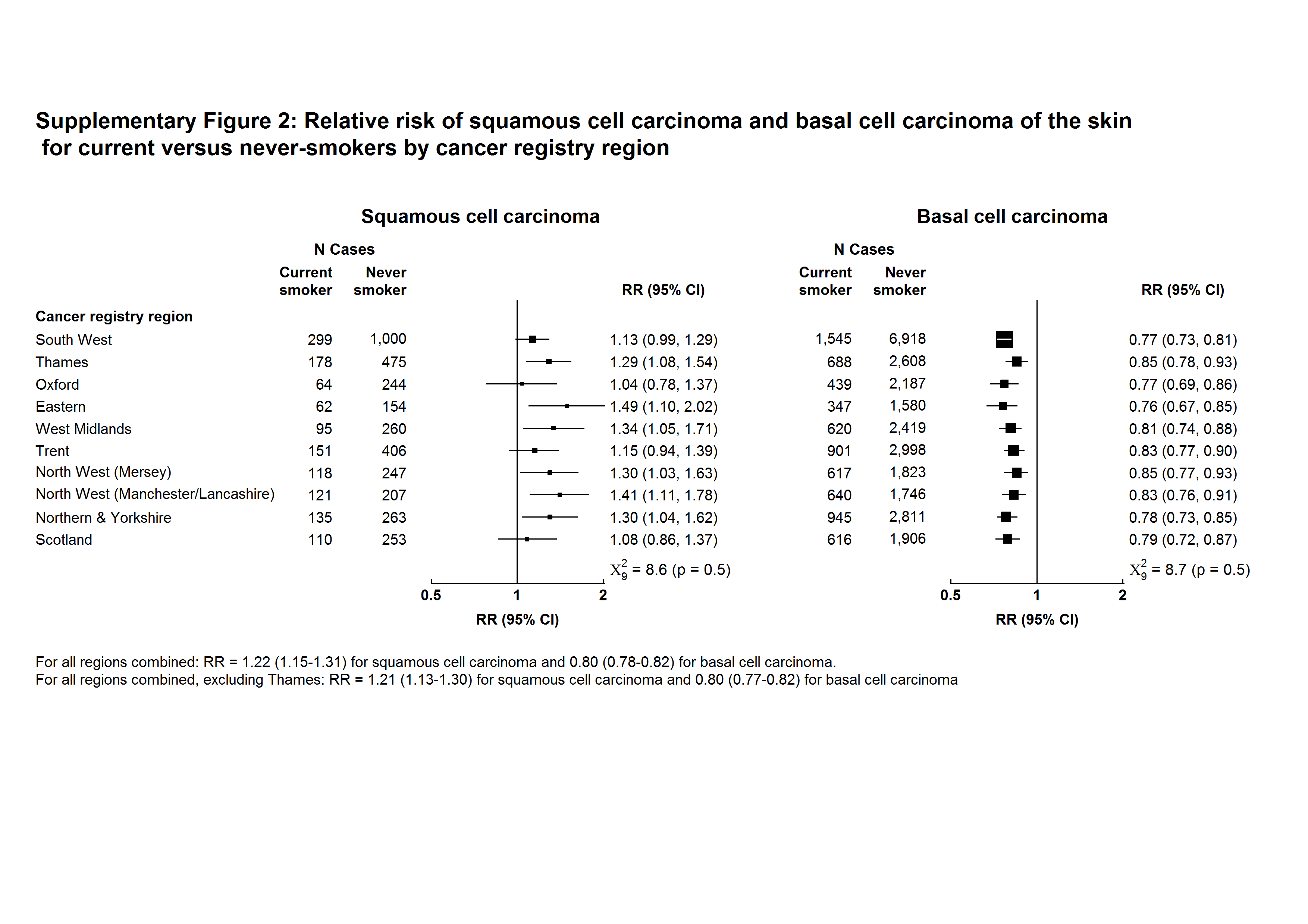
**

**Meta-analysis of prospective studies investigating the association between smoking and cutaneous squamous cell carcinoma (SCC) or basal cell carcinoma (BCC).**

**Methods:** A literature search was carried out in PubMed to identify prospective studies published from inception to 30 June 2017 that assessed the association between past smoking and current smoking compared with never smoking and the risk of SCC and/or BCC of the skin. The following search terms were used: (“carcinoma, squamous cell”[MeSH] OR “squamous cell carcinoma”[Title/Abstract] OR “carcinoma, basal cell”[MeSH] OR “basal cell carcinoma”[Title/Abstract] OR “nonmelanoma”[Title/Abstract] OR “non-melanoma”[Title/Abstract] OR “SCC”[Title/Abstract] OR “BCC”[Title/Abstract]) AND (“skin cancer”

[Title/Abstract] OR “cutaneous”[Title/Abstract] OR “skin neoplasms”[MeSH] OR “skin neoplasm*”

[Title/Abstract]) AND (“smoking”[MeSH] OR “smoke”[Title/Abstract] OR “smoking”[Title/Abstract] OR “tobacco”[Title/Abstract]) AND (“cohort studies”[MeSH] OR “prospective studies”[MeSH] OR “follow-up studies”[MeSH] OR “cohort”[Title/Abstract] OR “follow-up”[Title/Abstract] OR “follow up”[Title/Abstract] OR “prospective”[Title/Abstract] OR “prospectively”[Title/Abstract] OR “datalink*”[Title/Abstract]) NOT (“case reports”[Publication Type] OR “editorial”[Publication Type] OR “letter”[Publication Type] OR “comment”

[Publication Type] OR “case control studies”[MeSH] OR “treatment*”[Title/Abstract]).

Reference lists of identified studies were hand searched for further references.

Inclusion criteria:

- Prospective ascertainment of exposure
- Outcome of interest was incident cutaneous SCC and/or incident cutaneous BCC
- Current and past smoking versus never smoking were investigated as exposures
- The study reported HR or RR estimates (or equivalent) with 95% confidence intervals

Exclusion criteria:

- For cohort studies reported in multiple papers, publications with fewer cases were excluded
- Studies involving specific patient populations or participants with prior SCC or BCC of the skin
- Studies which only reported associations for ever versus never smoking

**Supplementary Figure 3.** Selection of studies for meta-analyses of the association between smoking and risk of cutaneous SCC and BCC.

64 articles excluded based on titles and abstracts:

- Cutaneous SCC or BCC were not outcomes of interest (n = 38)
- Smoking was not the exposure (n = 22)
- Studies in patient populations (n = 2)
- Review (n=2)

PubMed search (n=74)

Potentially relevant articles (n=10)

4 articles did not meet the inclusion criteria:

- Results were reported in a more recent paper (n = 3)
- Participants had prior cutaneous SCC or BCC (n = 1)

1 article added from reviews and other published articles

Articles included in meta-analyses (n=7)

**Supplementary Table 3.** Summary of prospective studies investigating the association between current and past smoking and cutaneous squamous cell carcinoma (SCC) and/or cutaneous basal cell carcinoma (BCC).

| **Cohort name, country and size** | **Publication (author, year) and follow-up period** | **Cancer ascertainment** | **Adjustment or stratification variables** | **SCC**  **Number of cases and RR (95% CI)** | **BCC**  **Number of cases and RR (95% CI)** |
| --- | --- | --- | --- | --- | --- |
| United States Radiological Technologists, USA  [n=68,371] | Freedman *et al.*, 2003  1983-1998 | Self-reported; validated by medical records | Age, sex, alcohol consumption, skin pigmentation, hair colour, race, education, body mass index, decade began employment as a radiation technologist, and proxy measures for residential childhood and adult sunlight exposure | Not reported | **Total cases=1,360**  **Current** (n=287)  RR=0.90 (0.80-1.00)  **Past** (n=458)  RR=1.10 (1.00-1.30) |
| Swedish construction workers, Sweden  [n=337,311] | Odenbro *et al.*, 2005  1971-2000 | Cancer registry | Age  Note: tobacco smoking included cigarette smoking, snuff dipping, cigar smoking and pipe smoking. | **Total cases=756**  **Current** (n=245)  RR=0.97 (0.80-1.17)  **Past** (n=141)  RR=0.95 (0.77-1.18) | Not reported |
| Nambour Skin Cancer Study,  Australia  [SCC: n=1,287]  [BCC: n=1,277] | McBride *et al.*, 2011 (SCC)  1992-2007  Hughes *et al.,* 2014 (BCC)  1992-2007 | Histologically confirmed through pathology records | SCC: Age, sex, skin colour, sun exposure, trial sunscreen treatment, and NSAID use (excluding SCC of lip)  BCC: Age, sex, country of birth, education, skin colour, trial sunscreen treatment, trial beta-carotene treatment, occupational sun exposure at baseline and post-trial sun exposure | **Total cases=188**  **Current** (n=16)  RR=1.11 (0.65-1.52)  **Past** (n=72)  RR=1.12 (0.82-1.50) | **Total cases=281**  **Current** (n=22)  RR=0.69 (0.45-1.05)  **Past** (n=95)  RR=1.05 (0.84-1.31) |
| Health Professionals Follow-up Study, USA  [n=44,799] | Song *et al.*, 2012  1986-2008 | Self-reported; validated by medical records for SCC | Body mass index, physical activity, history of cardiovascular diseases, type 2 diabetes, hypertension, hypercholesterolaemia and non-skin cancer, childhood reaction to sun, severe sunburns, moles, hair colour, family history of melanoma, sun exposures at different age intervals and UV index at birth, age 15 and age 30 years | **Total cases=991**  **Current** (n=67)  RR=1.31 (1.01-1.70)  **Past** (n=484)  RR=0.93 (0.82-1.06) | **Total cases=8,189**  **Current** (n=430)  RR= 0.85 (0.77-0.94)  **Past** (n=4,047)  RR=0.97 (0.93-1.02) |
| Nurses’ Health Study, USA  [n=100,910] | Song *et al.*, 2012  1984-2008 | Self-reported; validated by medical records for SCC | Body mass index, physical activity, history of cardiovascular diseases, type 2 diabetes, hypertension, hypercholesterolaemia and non-skin cancer, childhood reaction to sun, severe sunburns, moles, hair colour, family history of melanoma, sun exposures at different age intervals and UV index at birth, age 15 and age 30 years | **Total cases=1,339**  **Current** (n=182)  RR=1.38 (1.16-1.64)  **Past** (n=596)  RR=1.11 (0.98-1.24) | **Total cases=20,610**  **Current** (n=2,724)  RR=1.00 (0.95-1.04)  **Past** (n=8,871)  RR=1.08 (1.05-1.11) |
| Clinical Practice Research Datalink, UK  [n=114,242] | Reinau *et al.*, 2014  2000-2011 | Medical records | Alcohol status, body mass index, and number of general practitioner visits in the year before BCC diagnosis | Not reported | **Total cases=57,121**  **Current** (n=6,999)  RR=0.77 (0.74-0.80)  **Past** (n=19,607)  RR=0.91 (0.89-0.94) |
| QSkin Sun and Health Study, Australia  [n=18,828] | Dusingize *et al.*,  2017  2011-2014 | Histologically confirmed through pathology records | Age, sex, private health insurance, education status, natural skin colour, tanning ability, number of freckles, history of sunburn as a child, and cumulative sun exposure | **Total cases=193**  **Current** (n=34)  RR=2.30 (1.46-3.62)  **Past** (n=73)  RR=1.05 (0.74-1.48) | **Total cases=640**  **Current** (n=38)  RR=0.64 (0.44-0.93)  **Past** (n=217)  RR=0.85 (0.71-1.03) |
| Million Women Study, UK  [n=1,223,626] | Pirie *et al.*, 2018  1996-2014 | Cancer registry | Year of birth, year of recruitment, geographical region, socio-economic status, alcohol intake, strenuous physical activity, height, and body mass index | **Total cases=6,699**  **Current** (n=1,333)  RR=1.22 (1.15-1.31)  **Past** (n=1,857)  RR=1.00 (0.95-1.06) | **Total cases=48,666**  **Current** (n=7,358)  RR=0.80 (0.78-0.82)  **Past** (n=14,312)  RR=0.99 (0.97-1.01) |

**
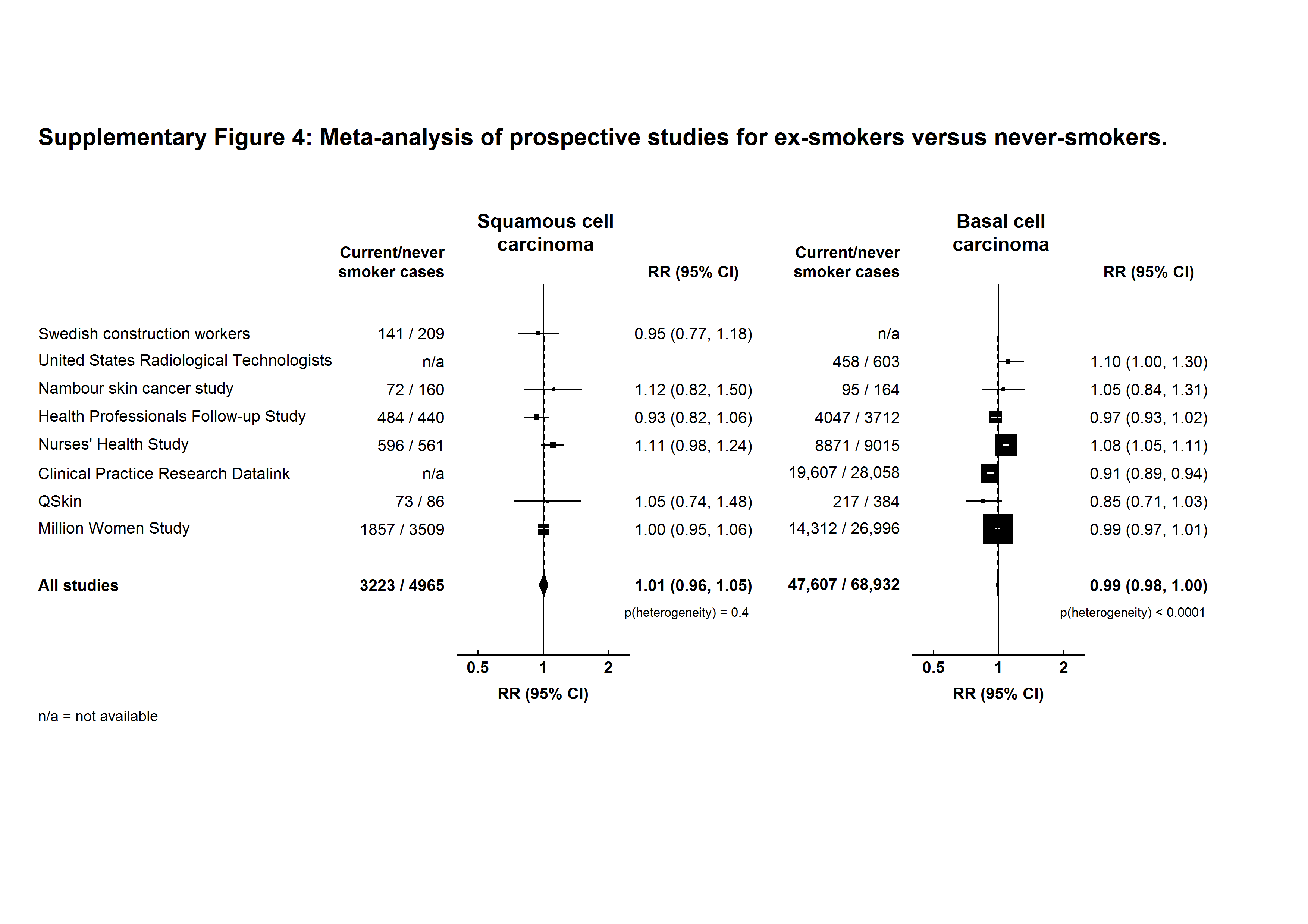
**
